# Genome-wide selection signal analysis reveals copy number variation associated with litter size in Guizhou Black goat

**DOI:** 10.3389/fvets.2025.1573093

**Published:** 2025-08-13

**Authors:** Yanpin Zhao, Yong Han, Chao Yuan, Yang Yang, Yong Long, Wen Xiao

**Affiliations:** ^1^Guizhou University of Traditional Chinese Medicine, Guiyang, China; ^2^Institute of Animal Husbandry and Veterinary Sciences, Guizhou Academy of Agricultural Sciences, Guiyang, China; ^3^College of Animal Science, Guizhou University, Guiyang, China

**Keywords:** CNV, goats, litter size, whole genome sequencing, select signal

## Abstract

**Introduction:**

Copy number variation (CNV) is a common form of genomic structural variation arising from genome sequence rearrangements, which primarily involves variations in the copy number of large genomic segments. This study focuses on performing selection signal analysis of CNVs to identify candidate loci and genes associated with litter size in Guizhou Black goat.

**Methods:**

Sample selection: 30 Guizhou Black goats were divided into high-lambing and low-lambing groups based on their continuous lambing records. Genetic analysis: Phylogenetic tree construction and principal component analysis (PCA) were performed to assess genetic differences between the two groups. Selection signal indicators: Genetic differentiation index (FST) and nucleotide diversity (θπ) were used as indicators for identifying selection signals. CNV identification: 180 CNVs associated with litter size were identified through selection signal analysis. Functional annotation: Candidate genes within these CNVs were annotated, and further analysis was conducted using Gene Ontology (GO) and KEGG pathway enrichment methods.

**Results:**

Genetic differentiation: Phylogenetic tree construction and PCA revealed significant differences in genetic structure between the high-lambing and low-lambing groups. CNV identification: 180 CNVs were found to be associated with litter size. Candidate genes: Functional annotation of the CNVs identified 49 candidate genes. Pathway enrichment: GO and KEGG pathway analysis revealed significant enrichment of these genes in several reproduction-related biological pathways, including the Hippo signaling pathway, steroid hormone biosynthesis, and retinol metabolism.

**Discussion:**

This study provides novel insights into the genetic mechanisms underlying prolificacy in Guizhou Black goats. The identification of CNVs associated with litter size offers potential targets for genomic selection breeding, which could be used to improve reproductive traits in this breed. The enriched pathways, such as the Hippo signaling pathway, steroid hormone biosynthesis, and retinol metabolism, suggest that these biological processes may play a critical role in regulating fertility and reproductive efficiency in goats. The findings highlight the potential for utilizing CNVs as markers for improving reproductive performance in livestock breeding programs.

## 1 Introduction

As economically significant livestock, goats contribute substantially to the global production of meat, milk, and hides. Increasing production volume alone is insufficient to meet the growing global demand for goat-derived products. Enhancing goat reproductive performance is a pressing concern, as litter size is a critical trait influencing reproductive efficiency and overall productivity. Consequently, enhancing litter size has become a primary objective in goat breeding programs. While factors such as maternal nutrition and dietary supplements (e.g., folic acid) play roles in determining litter size, genetic factors are also crucial. Recently, many studies have focused on identifying genetic variants and genes associated with litter size in goats. For instance, a genome-wide association study (GWAS) identified single nucleotide polymorphism (SNP) variants associated with litter size at birth (LSB) in Markhoz goats. Candidate genes annotated from these variants include GABRA5, AKAP13, and SV2B (1). Similarly, when analyzing SNPs associated with litter size in Chuanzhong Black goats, Guo et al. utilized FST and θπ analyses to examine population selection signals. Their findings annotated 506 and 528 genes in high-litter and low-litter groups, respectively. Additionally, polymorphism analysis revealed a significant association between the g.89172108 T > G variant in the exon of the AMH gene and litter size, suggesting its potential as a marker for selecting high-fertility individuals (2). As a complex trait, litter size is influenced by multiple genetic factors. Focusing exclusively on SNPs may not provide a comprehensive understanding of their genetic basis. Copy number variation (CNV), an essential form of structural variation involving changes in the copy number of large genomic segments, can significantly impact gene expression and function. Increasing evidence indicates that CNVs play a vital role in animal reproduction. For example, a CNV-based GWAS in Canadian Holstein cattle identified two CNVRs linked to three reproductive traits: calf survival rate, interval from first service to conception, and non-return rate (3).

The Guizhou Black Goat is a local meat-type breed native to the southwestern region of Guizhou Province, China. It is characterized by a moderate body size, tender meat with a mild odor, and strong adaptability to cold climates and low-quality forage. However, its relatively low reproductive performance has become a limiting factor for the breed’s development and widespread utilization. This study aims to identify copy number variations (CNVs) associated with lambing traits by analyzing whole-genome sequencing data of high- and low-litter-size Guizhou Black goat groups. We conducted selection signal analysis of CNVs between these groups to identify candidate genes potentially linked to litter size. These genes contribute to elucidating the genetic mechanisms underlying lambing traits and serve as valuable molecular markers for genetic improvement and enhanced reproductive performance in goats.

## 2 Materials and methods

### 2.1 Sample collection and whole genome resequencing

The 30 Guizhou Black goats used in this study were all sourced from Maiping Town, Huaxi District, Guizhou Province. The selected goats were healthy females aged 2–3 years, raised under uniform management conditions, and underwent annual disease screening. All experimental goats were fed a semi-grazing system, provided with the same basic diet (including silage corn, hay and concentrate) during the dry season, and allowed to eat natural pasture freely during the green season. Based on lambing records from the past 2 years, 15 high-litter-size individuals (average litter size ≥ 2) were selected to form the high-lambing group (HL), while 15 low-litter-size individuals (average litter size = 1) were chosen as the low-lambing group (LL) for whole-genome resequencing. Blood samples were collected using vacuum blood collection tubes between 8:00 and 9:00 in the morning. The studies involving animals were reviewed and approved by Guizhou University Subcommittee of Experimental Animal Ethics [eae-gzu-2024-e054]. Blood samples were collected for DNA extraction, and high-quality DNA samples were subsequently used to construct sequencing libraries. The DNBSEQ-T7 sequencer was employed to sequence, producing raw sequencing data with an average coverage depth of 33.3X.

### 2.2 CNV detection and annotation

To minimize errors caused by human factors during sequencing, the raw data were first filtered using Fastp (v0.23.4), and reads containing adapters were removed based on the following criteria. Paired reads were discarded if the proportion of “N” bases in a sequencing read exceeded 1% of the total bases. Additionally, paired reads were excluded if the number of low-quality bases (Q ≤ 5) in a sequencing read exceeded 50% of the total bases. Subsequently, BWA (v0.7.17) was used to align the clean reads to the reference genome (GCF_001704415.2_ARS1.2_genomic.chr), and SAMtools (v1.17) was employed to calculate basic metrics, such as sequencing depth and genome coverage, to prepare for subsequent variant detection. For structural variant (SV) detection, Manta was used to identify SVs for each individual. SVs of the same type were merged across individuals based on genomic position and variant length to remove redundancy. Paragraph genotyping was employed to genotype each individual using the deduplicated SV results. At the population level, further deduplication was performed based on positional overlap criteria (50% for deletions; 90% for insertions, inversions, and duplications) and genotype consistency (population genotype consistency ≥ 0.95).

Quality control was conducted on the deduplicated SVs using the following filtering criteria: variant length (ABS (INFO/SVLEN) ≤ 10,000,000), allelic frequency heterozygosity (INFO/ExcHet) ≥ 0.05, missing rate (F_MISSING) ≤ 0.2, and minor allele frequency (INFO/MAF) > 0. The Plink software was used to calculate CNV frequencies across all individuals, and R (v4.2.0) was used for visualization. Custom Perl scripts were employed for CNV analyses specific to different groups. Finally, all CNVs were annotated using Annovar.

### 2.3 Population structure analysis and selective feature detection

The experimental animals were divided into high-litter-size (HL, *n* = 15) and low-litter-size (LL, *n* = 15) groups based on their lambing records. Selection signature detection was then performed on these two groups. To understand the genetic structure of the HL and LL groups, principal component analysis (PCA) was conducted using the GCTA software (4) with the make-grm and pca commands. The PCA results were visualized using R software.

Next, two methods, genetic differentiation index (FST) and nucleotide diversity (θπ), were employed for selection signature analysis using VCFtools (v0.1.17). For FST analysis, a sliding window approach was applied with a 100 kb chromosome interval and a 10 kb step size. Subsequently, nucleotide diversity (*π*) values were calculated separately for each group, and the relative difference in nucleotide diversity between the groups was determined as log_2_(πHL/πLL). Candidate loci were identified by selecting those present in the top 1% of windows from both the FST sliding window analysis and the log_2_(πHL/πLL) sliding window analysis.

### 2.4 Functional annotation and pathway enrichment of candidate gene

The resulting data were filtered further after identifying candidate CNV loci within the selected regions based on the top 1% of FST sliding window analysis and log_2_(πHL/πLL). Goat gene annotation files were downloaded from the ENSEMBL database, and bedtools was used to annotate the candidate CNVs to their corresponding genes. Loci that were not enriched for genes or had negative FST values were excluded. Differential gene sets were then subjected to Gene Ontology (GO) and KEGG pathway enrichment analysis using the clusterProfiler package in R (v4.4.0). This analysis aimed to identify significantly enriched biological processes and pathways associated with the candidate CNVs.

## 3 Results

### 3.1 Identification and distribution of CNVs

**Figure 1:**
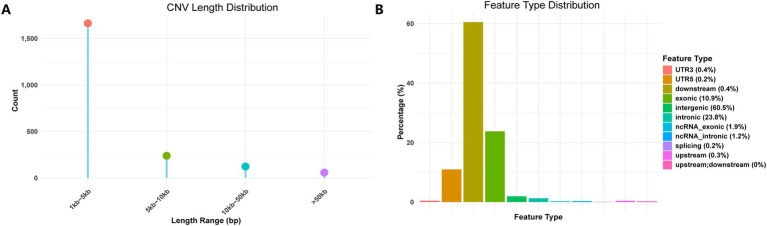
Identification statistics of CNVs. **(A)** CNV depth distribution. **(B)** Feature type distribution.

Following variant detection and filtering, a total of 2,083 CNV sites were retained. All detected CNVs were deletions, and the identification statistics are presented in Figure 1. A length distribution analysis of all CNVs revealed that most loci fell within the 1–5 kb range. Functional annotation of CNV regions indicated that approximately 60.5% were located in intergenic regions, 23.8% in intronic regions, and only 10.9% in exonic regions. The proportions of CNVs in downstream (0.4%) and upstream (0.3%) regions were relatively low. CNVs related to non-coding RNA were also uncommon, with ncRNA exons accounting for 1.9% and ncRNA introns for 1.2%. Additionally, CNVs were identified in 3′ untranslated regions (3′ UTR, 0.4%), 5′ untranslated regions (5′ UTR, 0.2%), splicing sites (0.2%), and overlapping upstream and downstream regions (0.05%).

**Figure 2:**
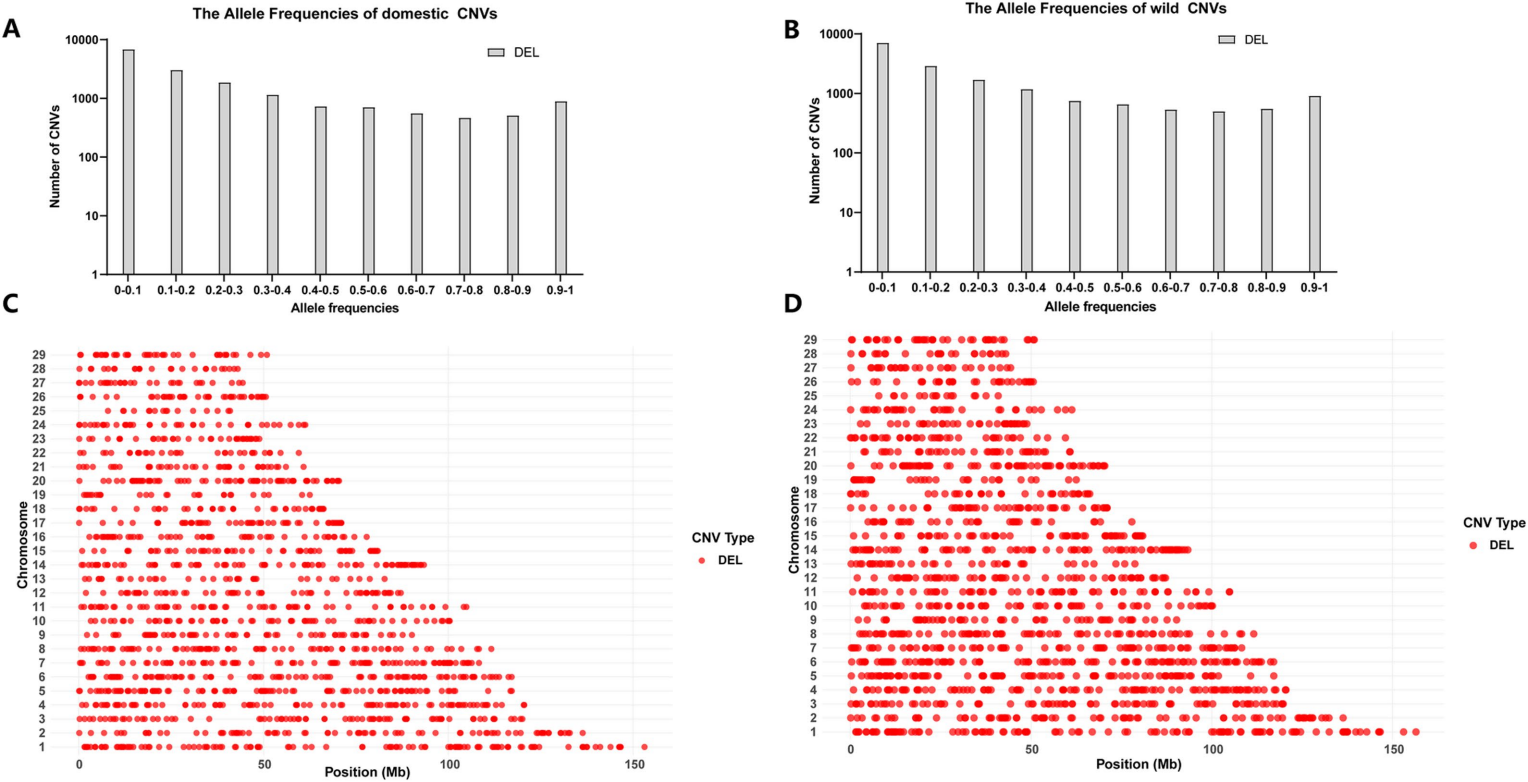
Distribution statistics of CNVs. **(A)** The number of CNVs at different frequencies in the HL group. **(B)** The number of CNVs at different frequencies in the LL group. **(C)** The distribution of chromosome CNVs in the HL group. **(D)** The distribution of chromosome CNVs in the LL group.

We analyzed the chromosomal distribution of CNVs and the number of CNVs at different frequencies in the HL and LL groups, and the results are shown in Figure 2. For statistical evaluation of the CNV distribution, we performed a chi-square test for the CNV distribution of each chromosome in both groups with a Bonferroni correction for multiple testing. The results, summarized in Supplementary Tables S1, S2, indicated no significant differences in CNV distribution at the chromosomal level between the HL and LL groups. Furthermore, the frequency statistics of CNVs showed no significant differences between the two groups. It is worth noting that, the results of Fisher’s exact test showed that the frequencies of deletion CNVs were significantly different between the HL and LL groups in the frequency ranges of 0–0.1 and 0.2–0.3.

### 3.2 Analysis of group structure

**Figure 3:**
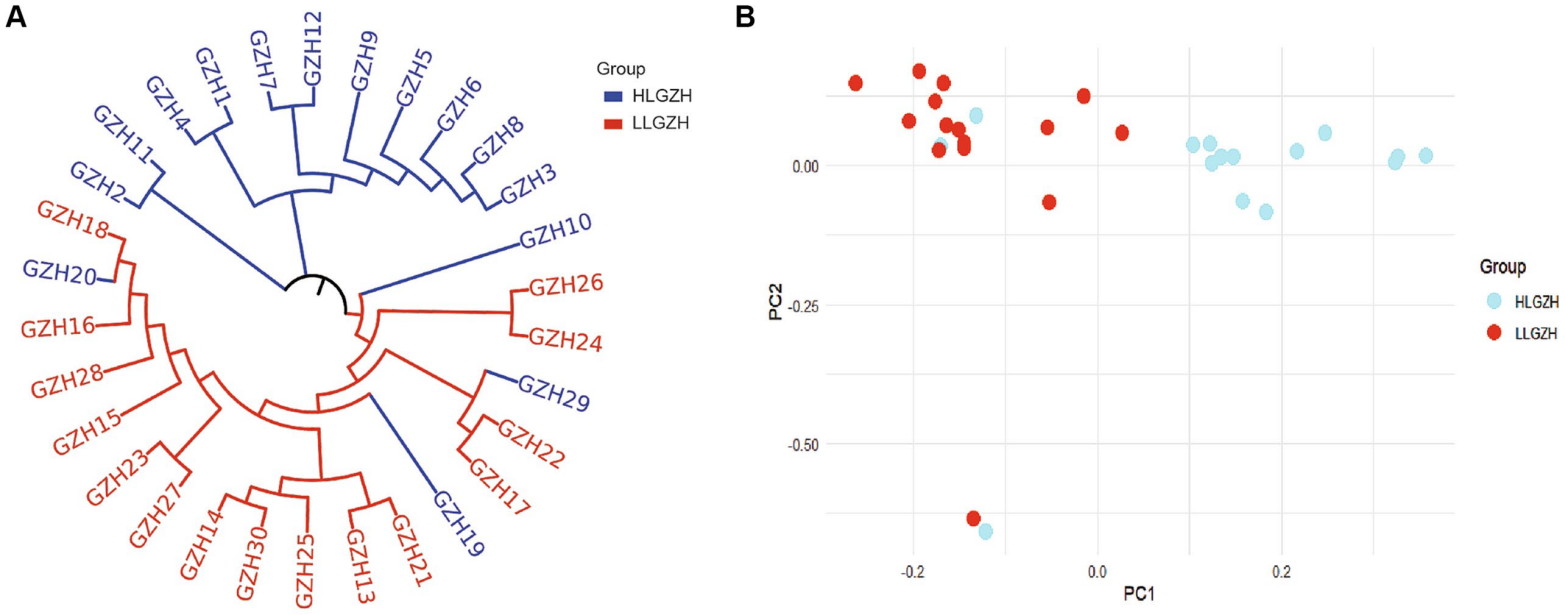
**(A)** Phylogenetic tree. **(B)** Principal component analysis.

The results of the principal component analysis (PCA) are presented in Figure 3. Despite some overlap between individuals from the HL and LL groups, the two groups display a distinct differentiation trend. This observation is corroborated by the phylogenetic tree in Figure 3, where all individuals are predominantly clustered into two separate branches, this also indicates that there is a clear differentiation between the two groups.

### 3.3 Selective signal analysis of HL and LL

**Figure 4:**
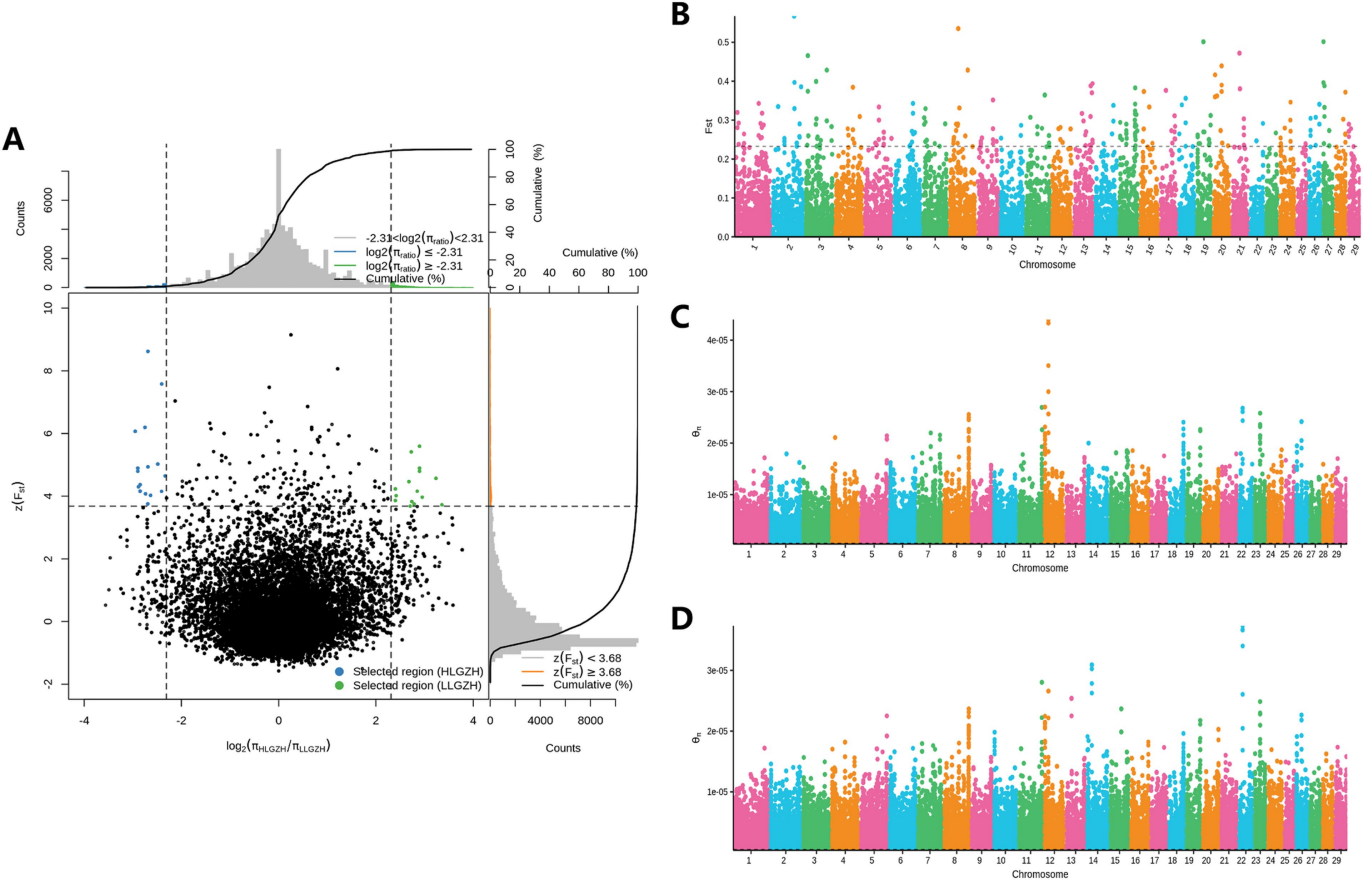
Selection of signal analysis. **(A)** Fst and log_2_(πHL/πLL) for the top 1%. **(B)** Manhattan plot of all CNVs (Fst). **(C)** Manhattan plot of CNVs (π) for the LL group. **(D)** Manhattan plot of CNVs (π) for the HL group.

The results of the selection signal analysis are presented in Figure 4. A total of 180 CNV loci were selected based on the relative difference in *π* values, log_2_(πHL/πLL), and the top 1% of FST. After annotation, 49 candidate genes (Table 1) were identified.

**Table 1:** Candidate gene functions (References (8–10, 21–48) are all listed in the table in top and bottom order).

Gene		Functionality	Reference
GJA4	Reproduction	Maturation and developmental competence of bovine oocytes	(8)
LINGO1		Bovine oocyte development	(9)
PTGER3		Promotes the onset of ovulation in monkeys	(10)
ZNF131		Inhibits estrogen signaling	(21)
MIR186		Regulates Follicle Stimulating Hormone (FSH) Expression	(22)
GJB3		Gene expression affects mouse embryo survival	(23)
HNF4A		Human Embryonic Stem Cell Differentiation	(24)
KDM4C		Mouse Embryonic Stem Cell Identity	(25)
PCDH15		Mouse Embryonic Development	(26)
SOX5		Involvement in mouse gonadal development	(27)
AXIN2		Human Embryonic Stem Cell Self-Renewal	(28)
STXBP6		Candidate Genes for Littering Traits in Pigs	(29)
TRNAC-GCA		Candidate genes for fertility in sheep	(7)
STK17B		Causes severe defects in porcine sperm acrosomes	(30)
CEP112		Coordinate spermatogenesis	(5)
DNAH7		Male reproductive disorders (sperm motility)	(32)
ALDH5A1	Disease	Good predictor of ovarian cancer	(33)
DGKE		Causes kidney related diseases	(34)
DLGAP3		Brain disorders	(35)
GPLD1		New therapeutic target for chronic diseases	(36)
PPP2R2B		Inhibitors of Triple Negative Breast Cancer (TNBC)	(37)
SERINC3		Limits HIV-1 Infectivity	(38)
TRNAG-CCC		Involved in inflammatory process of mastitis	(39)
TTPAL		Promotes gastric tumorigenesis	(40)
UTRN		Potential Biomarkers for Breast Cancer	(41)
ZNF438		Polycystic Ovary Syndrome (PCOS) Candidate Genes	(42)
ZRANB2		Involved in Adriamycin Resistance in Breast Cancer Cells	(43)
BET1	Other	Regulates protein transport between the endoplasmic reticulum and the Golgi apparatus	(44)
GJB4, GJB5		Mouse Epididymis Development	(45)
KIAA0319		Human neuroepithelial cell development	(46)
PKIG		Osteoblast differentiation	(47)
R3HDML		Satellite cell proliferation and differentiation	(48)
C19H17orf67, LOC102168522, LOC102179380, LOC102180243, LOC102180519, LOC102180784, LOC102183140, LOC102187692, LOC102188962, LOC102189228, LOC102189502, LOC102189778, LOC106501965, LOC108633190, LOC108638409, SMIM12			NA

### 3.4 GO and KEGG enrichment analysis of candidate genes

**Figure 5:**
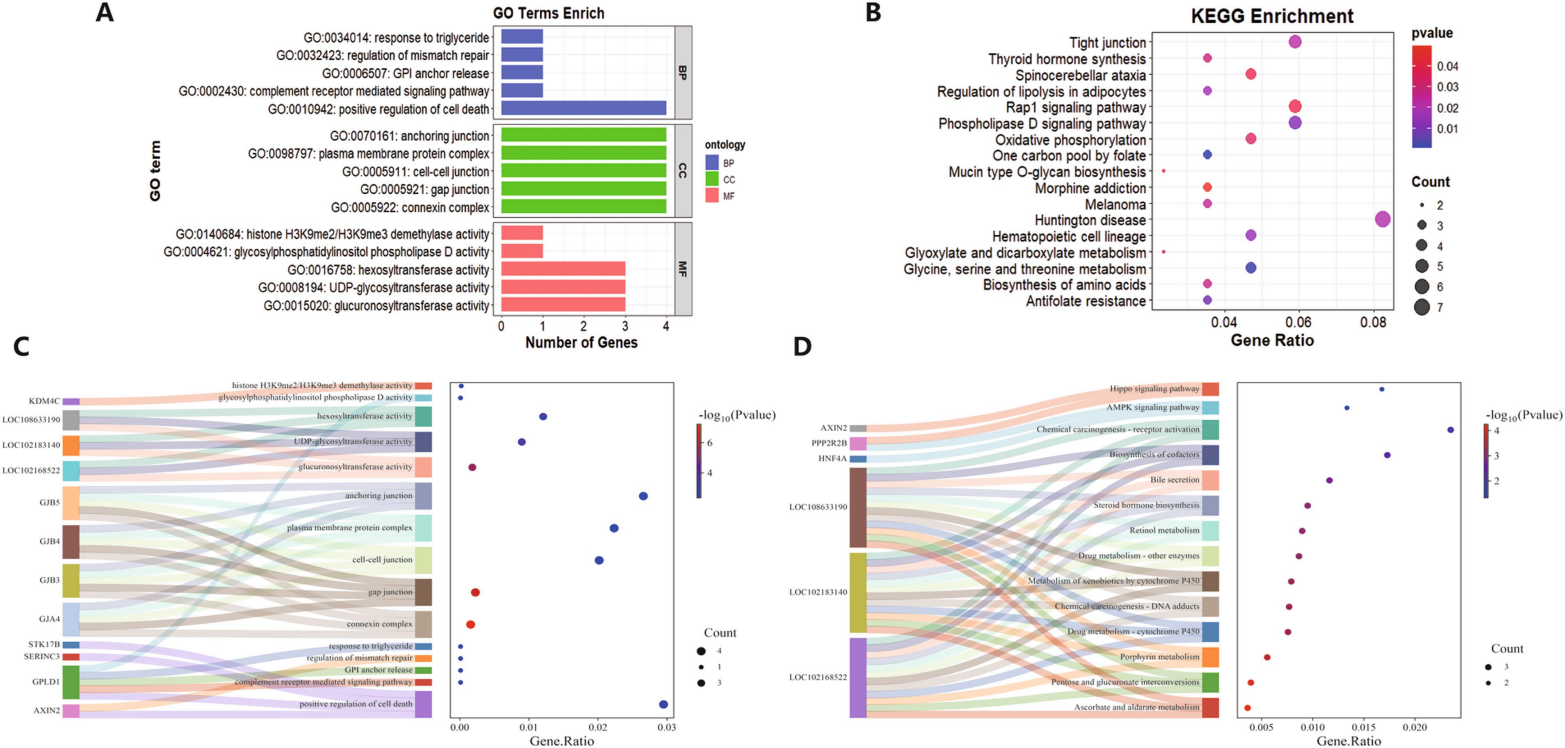
GO and KEGG enrichment. **(A)** GO-enriched PB, CC, and MF top 5. **(B)** KEGG top 17 significant pathways. **(C)** GO top 15 Sankey bubble plots. **(D)** KEGG top 14 Sankey bubble plots.

The primary significantly enriched pathways identified by GO and KEGG analyses are presented in Figure 5. A total of 59 KEGG pathways were enriched, including 14 significant pathways (*p* < 0.05), primarily associated with metabolism, hormones, signaling, and chemical carcinogenesis. The most significantly enriched pathway was ascorbate and aldate metabolism (chx00053). In the GO enrichment analysis, 532 pathways were identified, of which 134 were significant (*p* < 0.05). Among these, 453 biological processes (BP), 24 cellular components (CC), and 55 molecular functions (MF) were enriched, primarily related to apoptosis, stress response, lipid metabolism, development and regeneration, signal transduction, protein interactions, gene expression, and repair. The most significantly enriched biological process was positive regulation of cell death (GO:0010942), the most significantly enriched cellular component was connexin complex (GO:0005922), and the most significantly enriched molecular function was glucuronosyltransferase activity (GO:0015020).

## 4 Discussion

### 4.1 Distribution of CNV

We conducted a statistical analysis of the distribution and types of CNVs between the HL and LL populations. The results indicated that only deletion CNVs showed a significant difference, suggesting that the deletion copy number may be associated with lambing traits. Previous studies have demonstrated that CNV deletions are linked to animal reproductive performance. For example, Zhang Ruiqian (5) conducted a comparative analysis of CNVs in goats with different litter size trait groups. They found that in the deletion CNV analysis of high-yield and low-yield groups, 173 genes were unique to the high-yield group, indicating that the missing CNV may be related to the high litter size of goats. Similarly, Ting Sun et al. (6) found that a CNV deletion in SPAG16 was associated with reproductive ability (semen motility) in bulls.

### 4.2 Population structure analysis and selection signal analysis

The phylogenetic tree and principal component analysis results revealed clear genetic differentiation between the HL and LL groups, indicating structural genetic differences between the high-yield and low-yield groups, warranting further investigation. We reviewed the relevant literature and compiled statistics for the 49 selected genes (Table 1). Among them, 16 genes were classified as unknown, with no relevant studies identified. Of the remaining 33 genes, 16 have been identified in the literature as being associated with animal reproduction, while the others are linked to diseases, organ development, and cell differentiation. Notably, TRNAC-GCA was selected as a candidate gene for fertility in Katahdin ewes through whole-genome selection signal analysis (7), while STXBP6 was identified as a candidate gene in a meta-analysis of key SNPs related to pig litter size. Additionally, several candidate genes have been implicated in the regulation of ovulation-related processes in animals. For instance, GJA4 (8) supports oocyte maturation, while the roles of LINGO1 (9) and PTGER3 (10) appear more complex and may involve regulatory or inhibitory functions during follicular development and angiogenesis. Half of the 33 known candidate genes have been implicated in reproduction, further supporting the accuracy of the results in this study.

### 4.3 GO and KEGG enrichment

During the enrichment analysis, we identified three GO pathways directly related to placental development: “placenta development,” “embryonic placenta morphogenesis,” and “cell differentiation involved in embryonic placenta development.” Previous studies have shown that placental traits in sheep are associated with litter size (11), and alterations in litter size can lead to a reduction in placental weight (12). Abnormal placental development also can result in the death of some embryos, thereby reducing litter size. Furthermore, the placenta secretes various hormones that regulate pregnancy maintenance and fetal development, which influence litter size. For example, the placenta secretes progesterone (13), and research has shown that progesterone plays a role in determining bovine oocyte quality and embryo development (14). Oocyte quality and embryo development are closely linked to litter size. The two most significant GO terms in the enrichment analysis were “connexin complex” and “gap junction,” both of which are critical components of cell-to-cell communication and closely linked to reproductive function. Studies on gap junction proteins in the female reproductive organs of mice have shown that certain connexins (such as CX43, CX37, and CX26) are vital for female reproductive function (15).

In the KEGG enrichment analysis, we identified several pathways directly related to animal reproduction, such as the Hippo Signaling Pathway, Steroid Hormone Biosynthesis, and Retinol Metabolism. Studies have shown that the Hippo signaling pathway plays a critical role in ovarian development, follicular development, and oocyte maturation (16). Steroid hormones, including estrogen, progesterone, and testosterone, are directly involved in regulating the reproductive system and serve as core pathways for reproduction, controlling follicular development, ovulation, and pregnancy maintenance (17). Additionally, retinol (vitamin A) plays a vital role in the reproductive system, particularly in regulating embryonic development (18), follicular maturation, and spermatogenesis (19).

Notably, when we created Sankey bubble plots for the GO and KEGG enrichment results, we identified three genes common to both plots: LOC102168522, LOC102183140, and LOC108633190. A review of the relevant literature revealed that, while the overexpression of LOC102168552 has been shown to promote the growth of mammary epithelial cells (20), no studies have reported on the functional roles of the other two genes. We hypothesize that these three genes may be associated with lambing performance in Guizhou Black goats; however, definitive conclusions can only be drawn through further in-depth investigation of these genes.

## 5 Conclusion

In this study, we conducted a genome-wide selection signal analysis using the genetic differentiation index (Fst) and nucleotide diversity (θπ) to identify CNVs associated with lambing performance in Guizhou Black goats. A total of 2083 CNVs were detected in the HL and LL groups, and 180 CNVs were selected as potentially associated with lambing performance. Furthermore, we identified 49 candidate genes, including TRNAC-GCA, STXBP6, and GJA4, which may be linked to lambing traits.

## Data Availability

The original sequence data of Guizhou Black Goat used in this paper have been deposited in the National Genome Science Data Center (Nucleic Acids Research 2022) and are publicly accessible at the following URL: https://bigd.big.ac.cn/gsa/browse/CRA019130.
